# A Unique Presentation of a Rectosigmoid Intussusception in a 59-Year-Old Female: A Case Report

**DOI:** 10.7759/cureus.115179

**Published:** 2026-08-25

**Authors:** Riyad Hermas, Samson Chan

**Affiliations:** 1 Medicine, Saint James School of Medicine, Arnos Vale, VCT; 2 Surgery, Insight Hospital and Medical Center, Chicago, USA

**Keywords:** adult intussusception, case report, colorectal intussusception, diverting colostomy, gi oncology, hartmann procedure, intussusception in adults, intussusception lead point, left hemicolectomy, rectosigmoid intussusception

## Abstract

Adult intussusception is an uncommon condition that normally presents with nonspecific GI symptoms. It is typically associated with some form of a pathological lead point, especially malignancy in colonic cases. This report discusses the case of a 59-year-old female who presented with abdominal distention and constipation with on-and-off rectal bleeding for two days prior to ED admission. Imaging with computed tomography (CT) revealed findings compatible with a distal intussusception of the sigmoid colon/rectum. Emergent surgical intervention confirmed the diagnosis via a laparoscopic left-sided colectomy that required splenic flexure mobilization. Primary anastomosis was deferred due to heavy stool burden, and a Hartmann procedure was performed. Histopathological examination revealed a 3.0 × 2.5 × 2.2 cm well-differentiated adenocarcinoma infiltrating the muscularis propria. The resection specimen was staged as pT2N0Mx, with negative surgical margins.

This is a case of an unusual, self-resolving adult rectosigmoid intussusception with an obstructing malignant lead point. The patient's history and clinical presentation of intermittent abdominal pain, rectal bleeding, and constipation necessitate the need for imaging. CT, coupled with colonoscopy, provides the best chance for definitive diagnosis and guidance for possible treatment. Surgical intervention remains the first-line treatment for obstructing malignant lead points.

Adult rectosigmoid intussusception is a rare condition; its intermittent presentation and nonspecific symptoms make diagnosis difficult. Only by maintaining a high level of suspicion and applying appropriate imaging modalities can a definitive diagnosis be established. The Hartmann procedure is one of several surgical options for rectosigmoid intussusception with a malignant lead point.

## Introduction

Intussusception is a condition in which one segment of the bowel telescopes into an adjacent segment. This could resolve spontaneously or recur intermittently. It could also progress into painful bowel obstruction and lead to bowel wall ischemia and necrosis if it persists. The cause is typically an ulceration, a diverticulum, an adhesion, or a neoplasm that acts as a lead point pulling one segment of the bowel into another during peristalsis [1].

Whereas the etiology of child intussusception is mostly idiopathic, lacking an identifiable lead point, the association of adult intussusception with malignancy is present in more than 40% of adult cases [2]. The condition, therefore, affects far more children than adults, with a ratio of 20:1 [2]. Colorectal intussusception is far less likely than a small-intestine intussusception, accounting for only 19.9% of all adult intussusceptions [3]. Given the added complication of intermittent symptoms, the diagnosis of rectosigmoid intussusception in adults is a diagnostically challenging event. Consequently, it is usually not prioritized on the list of differential diagnoses that present with the nonspecific symptoms of abdominal pain, constipation, nausea, vomiting, diarrhea, or GI bleeding [1].

In this case presentation, the symptoms resulted from a rare self-resolving rectosigmoid intussusception instigated by a sigmoid malignancy.

This case report has been reported in line with the Surgical CAse REport (SCARE) checklist [4].

## Case presentation

This is the case of a 59-year-old female with a history of coronary artery disease (CAD) on aspirin and statin, pacemaker, hypertension on amlodipine, asthma on albuterol and Symbicort as needed, smoking and substance abuse, and a recent unintentional weight loss leading to a BMI of 16 kg/m2. The patient presented to the emergency department in July 2025 with lower abdominal pain, constipation, and a two-day history of copious amounts of bright red blood per rectum. A summary of the timeline of this presentation is included (Table 1).

**Table 1:** Timeline summary of presentation CEA - carcinoembryonic antigen; PET - positron emission tomography; ED - emergency department; CT - computed tomography

Date	Event/treatment
March 2025	Sigmoid mass identified with negative biopsy and CEA results
May 2025	PET scan showing avid intramural lesion in rectosigmoid colon
June 2025	Sigmoidoscopy
July 2025	ED presentation, CT scans, colonoscopy, surgery
September 2025	First follow-up visit to the surgery clinic
April 2026	Most recent follow-up visit to the surgery clinic

The patient reported similar symptoms that started more than six months prior. In March 2025, she was evaluated at another facility, where a sigmoid mass was identified; however, biopsy and carcinoembryonic antigen (CEA) results were negative. A subsequent PET scan in May 2025 showed a 2.9 cm avid intramural lesion in the rectosigmoid colon. A follow-up sigmoidoscopy in June 2025 revealed a partially obstructing, fungating, polypoid mass in the distal sigmoid colon. The mass was partially circumferential, involving one-half of the lumen circumference, and it measured 3 cm in length. The area was tattooed with 4 mL of Spot (carbon black). Biopsy findings indicated tubulovillous adenoma with high-grade dysplasia. The patient was referred to general surgery for resection, but the patient's condition deteriorated prior to follow-up.

**Figure 1:**
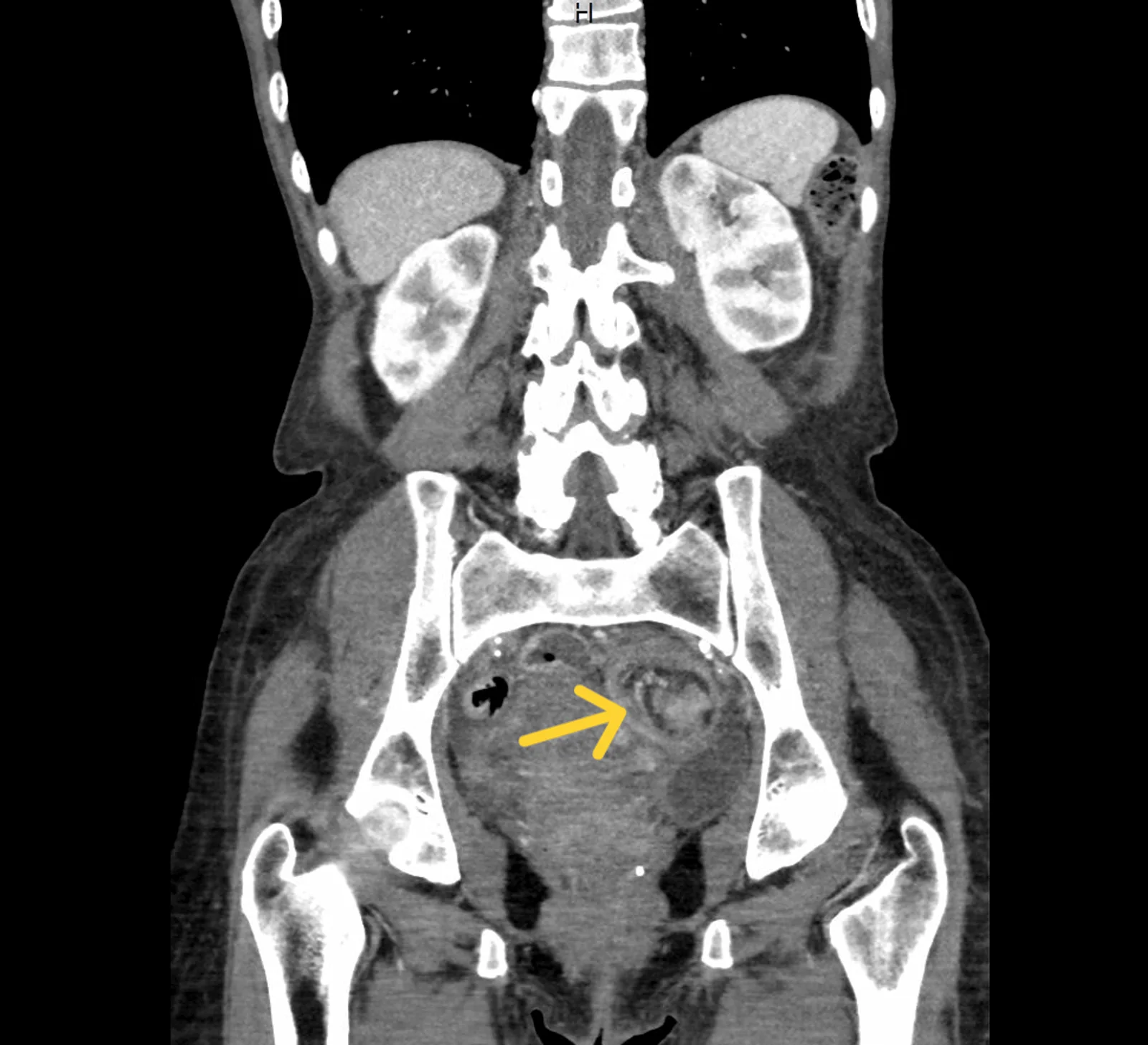
Target sign of rectosigmoid intussusception on CT

**Figure 2:**
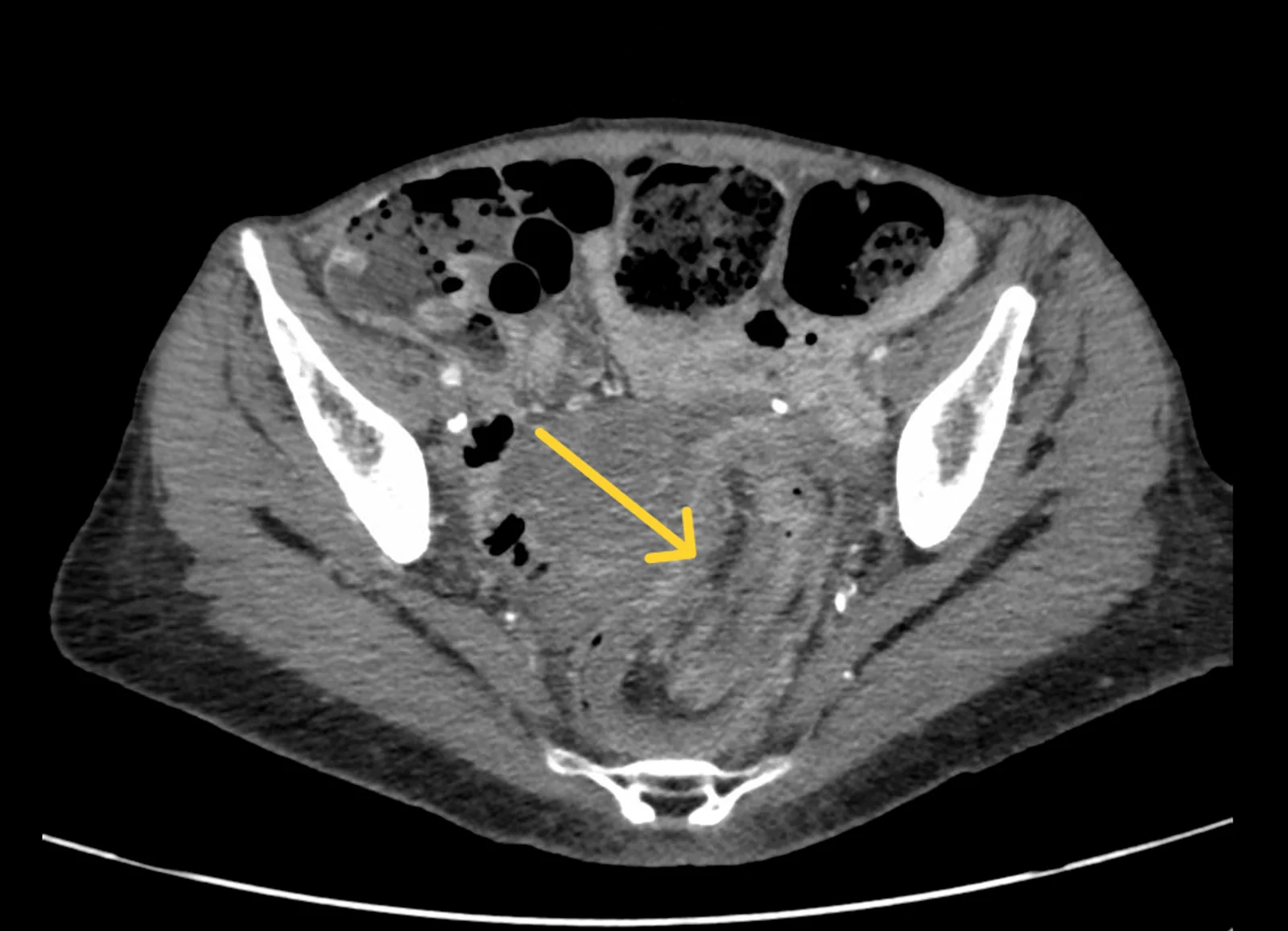
Bowel-in-bowel configuration of rectosigmoid intussusception on CT

The patient reported repeat episodes of abdominal cramping and bloody stool had occurred intermittently every two months, leading up to the current presentation on July 6th, 2025. In the ED, the physical exam was remarkable for a non-distended, non-tender abdomen with no guarding or rebound. Rectal exam showed no stool in the vault with scant mucus that was guaiac negative. The patient was tolerating oral intake and denied nausea or vomiting. Laboratory results were grossly normal, showing a hemoglobin of 14 gm/dL. A STAT abdominopelvic computed tomography (CT) with contrast captured a rectosigmoid intussusception. The CT scan was repeated without contrast, which confirmed the intussusception (Figure 1, Figure 2).

**Figure 3:**
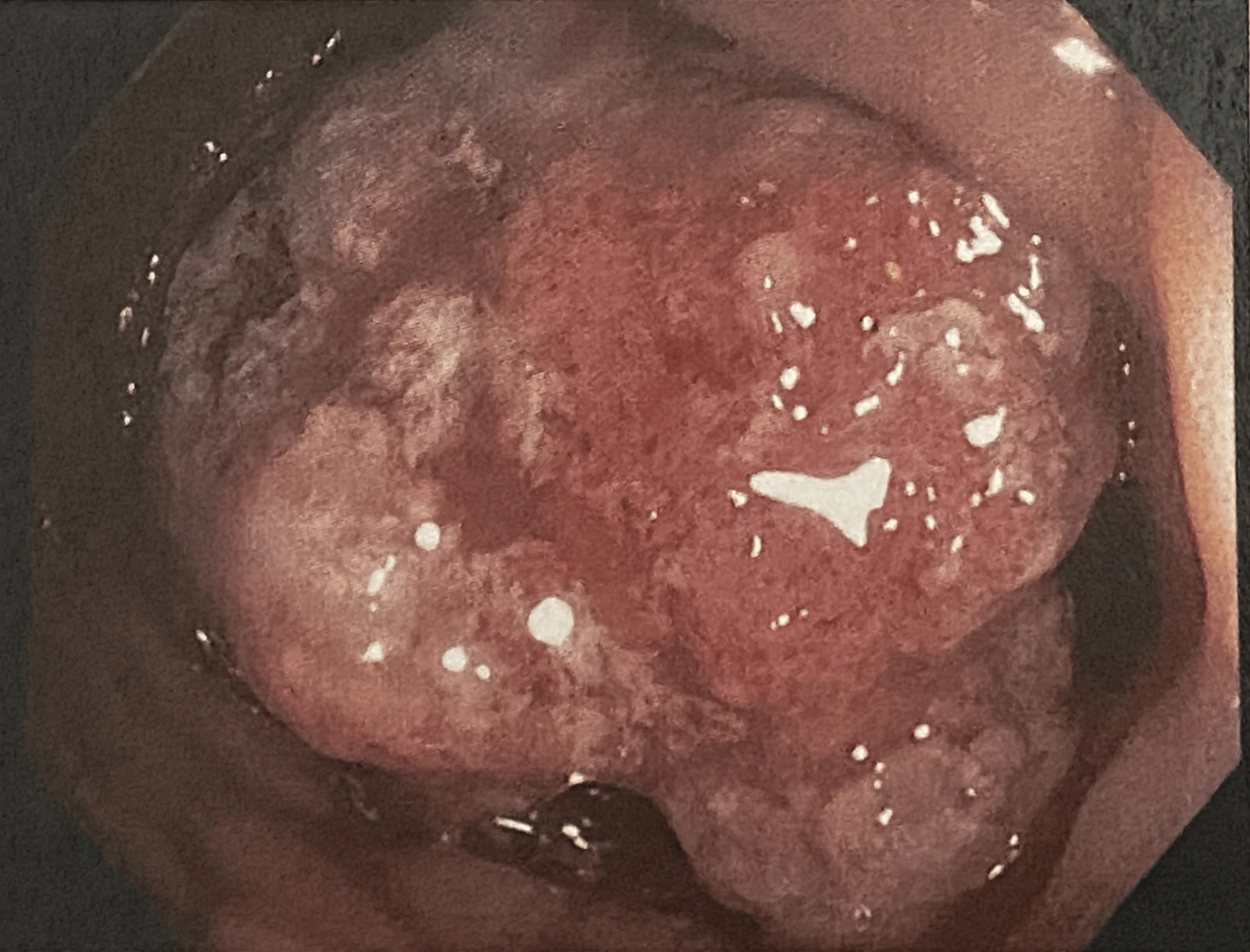
Mass obstructing the distal sigmoid

Further evaluation was done with a colonoscopy to rule out ischemia, obtain a biopsy, decompression, and possible reduction in preparation for surgery. Findings of this colonoscopy demonstrated intact viable bowel walls and a resolved intussusception. Additionally, a copious amount of stool was noted proximal to a nearly complete obstructing sigmoid mass (Figure 3).

The general surgeon on the case performed an emergent laparoscopic left hemicolectomy with end colostomy; primary anastomosis was deferred due to heavy stool burden. Following the establishment of general anesthesia, a 5 mm infraumbilical skin incision was made, and the abdomen was insufflated with low-flow CO_2_ to a steady pneumoperitoneum pressure of 15 mmHg. At this point, a 5 mm Visiport was inserted; under direct visualization, a 5 mm port was inserted in the upper abdomen, and two additional 5 mm ports were placed in the lower right mid-abdomen. The umbilical port was exchanged for a 12 mm port for planned use of an endoscopic stapler. Evaluation of intra-abdominal contents appeared to be grossly normal. The sigmoid tumor at the tattooed region was located about 25-30 cm from the anal verge without perforation or local invasion. In addition, multiple enlarged lymph nodes were palpated.

The inferior mesenteric artery and vein were clearly identified and dissected close to the take-off region from the aorta. These large vessels were isolated, dissected, triple-clipped, and divided at the base with Medtronic Ligasure to ensure adequate node retrieval. Splenic flexure mobilization was then performed by taking down the gastrocolic ligament, the splenocolic ligament, the phrenicocolic ligament, and the pancreaticocolic ligament with the Ligasure with minimal blood loss. The mesocolon was also separated from the retroperitoneum and from Gerota's fascia along the colon areas of interest from the descending colon and sigmoid to the transverse colon. The rectum was then transected using the Medtronic Endo-GIA (Medtronic, Galway, Ireland) medium-thick load linear stapler. Proximal transection was then performed using the same stapler load at the distal transverse colon. To retrieve the specimen, a periumbilical incision was made by extending the port site to the size of the mass. The proximal transected end of the colon was brought up through a left abdominal incision sized appropriately for a colostomy. The periumbilical midline defect made to retrieve the colon specimen was then closed in layers with Ethicon 0 polydioxanone (PDS) running suture (Johnson & Johnson, New Brunswick, New Jersey). The end colostomy was matured in the typical rosebud fashion using Ethicon 3-0 silk (Johnson & Johnson, New Brunswick, New Jersey) to invert the mucosa and anchor it to the fascia. Ethicon 3-0 Vicryl (Johnson & Johnson, New Brunswick, New Jersey) buried interrupted sutures were used to attach the colostomy to the dermis. Digital probe testing of the colostomy showed good patency. The port sites were closed with buried interrupted 3-0 Vicryl sutures. All subcutaneous tissue and skin were closed in layers. The surgery lasted four hours and 45 minutes, and the blood loss was estimated at 30 mL.

Histopathology revealed a well-differentiated 3.0 × 2.5 × 2.2 cm rectosigmoid adenocarcinoma infiltrating the muscularis propria with negative surgical margins and pT2N0Mx for staging. The 26 cm long specimen contained 14 lymph nodes, of which the largest node measured 1.2 cm; all were negative for metastatic disease. 

The patient had an uncomplicated postoperative course, and by postoperative day six, she was tolerating solid foods well and had regained bowel function in her colostomy, allowing discharge home with regular outpatient clinic follow-up. Two months later, the patient followed up with the surgeon and presented with improved health and appropriate weight gain. Subsequent reversal of her colostomy has not been performed yet due to the patient's social and familial obligations, for which she moved out of state for several months. She recently had a follow-up appointment with the surgery clinic. The patient felt and looked healthy and was ready for a colostomy reversal; however, she had no follow-up with her oncologist and no up-to-date PET scan or CEA level since the surgery. The patient was also informed that a follow-up colonoscopy will be needed to guide the decision to reverse the colostomy.

## Discussion

Adult rectosigmoid intussusception represents a rare subset of adult colorectal intussusceptions, which in turn account for less than 1% of all reported cases of intussusception. The intermittent nature of intussusception symptoms makes accurate early diagnosis challenging. Nevertheless, a thorough investigation of the underlying etiology of these symptoms can lead to early detection of malignancy. In this case report, the patient's initial presentation did not suggest intussusception, but it did indicate a mass with negative pathology. Subsequent presentations and further investigations demonstrated the importance of maintaining a high level of suspicion for malignancy-induced intussusception and of using appropriate diagnostic tools.

Early diagnosis relies heavily on accurate interpretation of radiologic findings to avoid discovery during surgical intervention [5]. On ultrasonography, characteristic features may include a "target sign" on transverse imaging and a "pseudo-kidney sign" on longitudinal imaging [6]. These signs are easily identified in the case of child intussusception, which tends to be benign. However, computed tomography, which typically demonstrates the classic "bowel-within-bowel" configuration, is the imaging of choice in adult intussusceptions, which have been shown to be associated with malignancy in more than 40% of the cases [2,7]. According to Kim et al., the presence of bowel-within-bowel on abdominal CT is pathognomonic of intussusception. An added advantage to CT imaging is the ability to distinguish between lead-point and non-lead-point intussusceptions, thereby reducing the number of unnecessary surgical interventions [8]. In this case, the bowel-within-bowel configuration is evident in Figure 2.

Despite the central role of imaging, invasive investigations such as colonoscopy and sigmoidoscopy remain useful both for diagnosis and for therapy. Diagnostically, direct visualization can help evaluate the underlying causes (i.e., leading point) of intussusceptions while providing a histology sample, thereby guiding the planning of further treatments [9]. Therapeutically, intussuscepted bowels can be manipulated and reduced during a colonoscopy. However, reduction should be avoided in the presence of a malignant lead point or signs of inflammation or ischemia of the bowel wall due to risks of: (1) intraluminal seeding and venous tumor dissemination, (2) perforation and seeding of microorganisms and tumor cells to the peritoneal cavity and (3) increased risk of anastomotic complications of the manipulated friable and edematous bowel tissue [10]. In this case, the intussusception had naturally resolved before the colonoscopy was done. This occurrence reaffirmed the intermittent history of this presentation, which is highly unusual, as the literature reports only self-resolving intussusceptions in relation to transient, non-obstructing, non-lead point intussusceptions.

In adult intussusception, the likelihood of an underlying malignant lead point is sufficiently high to justify routine surgical resection. Resection is also indicated when surgeons suspect bowel wall ischemia. Resection with primary anastomosis, primary anastomosis with proximal diverting loop ileostomy, or a Hartmann procedure can all be performed safely, depending on the surgeon's level of expertise, the presence of factors of anastomotic leak, and intraoperative field contamination. The Hartmann procedure is generally recommended for left-sided colonic intussusception [1]. The procedure is valued for its speed, simplicity, and effectiveness in mitigating the mortality and morbidity associated with emergency colorectal surgery [11]. The surgery can be open or laparoscopic, but laparoscopic resection is preferred due to quicker return of gastrointestinal function and shorter hospital stay [12]. In this presented case, the surgeon performed an emergent laparoscopic left colectomy with end colostomy and closure of the distal segment (i.e., Hartmann procedure); primary anastomosis was deferred due to heavy stool burden.

Given that histological analysis revealed a well-differentiated adenocarcinoma infiltrating into the muscularis propria with negative resection margins and pT2N0Mx for staging, the prognosis is favorable. According to Xu et al., the 10-year overall survival rate is greater than 71% for a 60-year-old female with T2N0M0 colorectal cancer [13]. The patient followed up with the surgeon two months after surgery, demonstrating well-healing incisions, a viable colostomy, improved health, and appropriate weight gain. Colostomy reversal is safe as early as 45 to 110 days after an uncomplicated Hartmann procedure, with improved outcomes in earlier reversal [14]. Nevertheless, socioeconomic constraints can affect reversal timing, as is the case with this patient, whose reversal has been deferred due to social and familial obligations. Once the patient is able, a follow-up colonoscopy is required prior to proceeding with reversal. Follow-up standards of care for such patients are based on a stage-dependent five-year surveillance period as outlined by the National Comprehensive Cancer Network (NCCN), the American Society of Colon and Rectal Surgeons (ASCRS), and the American College of Surgeons (ACS) guidelines.

## Conclusions

Adult rectosigmoid intussusception is a rare condition accounting for less than 1% of all intussusceptions; its intermittent presentation and nonspecific symptoms make diagnosis difficult. Only by maintaining a high level of suspicion and applying appropriate imaging modalities can a definitive diagnosis be established. Abdominopelvic CT is the gold standard for diagnosis, while a colonoscopy with biopsy can guide appropriate treatment by visualizing the underlying cause and determining histological characteristics of the bowel. In the case of a malignant lead point, surgical options for resection include primary anastomosis, primary anastomosis with proximal diverting loop ileostomy, or the Hartmann procedure.
